# Sexual and asexual oogenesis require the expression of unique and shared sets of genes in the insect *Acyrthosiphon pisum*

**DOI:** 10.1186/1471-2164-13-76

**Published:** 2012-02-15

**Authors:** Aurore Gallot, Shuji Shigenobu, Tomomi Hashiyama, Stéphanie Jaubert-Possamai, Denis Tagu

**Affiliations:** 1 INRA, UMR 1349 IGEPP, Institut de Génétique Environnement et Protection des Plantes, 35653 Le Rheu cedex, France; 2 Okazaki Institute for Integrative Bioscience, National Institute for Basic Biology, National Institutes of Natural Sciences, Higashiyama, Myodaiji, Okazaki, 444-8787, Japan

## Abstract

**Background:**

Although sexual reproduction is dominant within eukaryotes, asexual reproduction is widespread and has evolved independently as a derived trait in almost all major taxa. How asexuality evolved in sexual organisms is unclear. Aphids, such as *Acyrthosiphon pisum*, alternate between asexual and sexual reproductive means, as the production of parthenogenetic viviparous females or sexual oviparous females and males varies in response to seasonal photoperiodism. Consequently, sexual and asexual development in aphids can be analyzed simultaneously in genetically identical individuals.

**Results:**

We compared the transcriptomes of aphid embryos in the stages of development during which the trajectory of oogenesis is determined for producing sexual or asexual gametes. This study design aimed at identifying genes involved in the onset of the divergent mechanisms that result in the sexual or asexual phenotype. We detected 33 genes that were differentially transcribed in sexual and asexual embryos. Functional annotation by gene ontology (GO) showed a biological signature of oogenesis, cell cycle regulation, epigenetic regulation and RNA maturation. *In situ *hybridizations demonstrated that 16 of the differentially-transcribed genes were specifically expressed in germ cells and/or oocytes of asexual and/or sexual ovaries, and therefore may contribute to aphid oogenesis. We categorized these 16 genes by their transcription patterns in the two types of ovaries; they were: i) expressed during sexual and asexual oogenesis; ii) expressed during sexual and asexual oogenesis but with different localizations; or iii) expressed only during sexual or asexual oogenesis.

**Conclusions:**

Our results show that asexual and sexual oogenesis in aphids share common genetic programs but diverge by adapting specificities in their respective gene expression profiles in germ cells and oocytes.

## Background

Sexual reproduction involves two main events: meiosis and fertilization, and creates new genotypes by shuffling allelic combinations. Although the predominance of sexual reproduction in eukaryotes supports this innovation as a successful reproduction strategy, asexuality has evolved independently multiple times from sexual ancestors in almost all major taxa [1-4], such as in stick insects [3] and *Ranunculus *plants [4]. How asexuality has evolved in sexual organisms is unclear. In aphids, asexuality was acquired once about 250 million years ago by a common sexual ancestor [5]. Most aphid species alternate between sexual reproduction and asexual parthenogenetic reproduction according to seasonal variations. In spring and summer, aphids reproduce asexually by parthenogenesis and produce clonal parthenogenetic female progeny by viviparity. The autumnal shortening of the photoperiod induces the concentration of juvenile hormone (JH) to decrease in the aphid haemolymph., and particular form of parthenogenetic female called the sexuparae are produced. Sexuparae females produce sexual females and males that subsequently mate to produce overwintering eggs. Although parthenogenetic viviparous females and sexual oviparous females exhibit major differences in morphology and behavior, they share the same genome. This phenomenon, called reproductive polyphenism, is an example of aphid phenotypic plasticity [6].

The cellular and cytogenetic bases of reproductive polyphenism have been described for several aphid species [7,8]. In aphids, three generations are represented within one viviparous female: the mature embryos developing inside the maternal abdomen carry the first developmental stage of the third generation. This phenomenon is known as the 'telescoping of generations' [9]. The embryonic developments of asexual and sexual females are similar until the formation of undifferentiated germaria, which takes place at the end of germ band retraction (stage 18) [8]. The 32 germ cells within the germaria enter into early meiotic prophase (zygotene) and a partial association between bivalents occurs [7]. After this stage, the embryonic development and maturation of germ cells diverge between sexual and asexual females. Ancestral sexual development occurs under short photoperiod: the basal cells of germaria progress through meiosis; the sexual oocyte displays chromosome pairing, synapsis and meiotic recombinations. Meiosis is blocked in first meiotic prophase until the egg is fertilized and laid. Under long photoperiod, sexual oogenesis is inhibited [7]. The asexual oocyte ovulates in the posterior part of the germaria and forms a new follicle where it skips the first meiotic division and undergoes a single maturation division resulting in a discarded polar body and a diploid clonal oocyte. Synchronous mitotic divisions follow immediately without fertilization to initiate early embryogenesis, which continues within the parthenogenetic female abdomen and progresses through a series of 20 developmental stages [8].

We identified genes regulated in sexual and asexual aphid oogenesis by combining transcriptomic analyses and *in situ *hybridizations. In aphids, sexual and asexual reproduction can be analyzed in individuals possessing identical genomes. Sequencing of the LSR1 pea aphid genome [10] and the recent development of new genomic resources offer an unique opportunity to understand the molecular bases of sexual and asexual reproduction. Our results demonstrate that sexual and asexual reproduction pathways require unique and shared genetic programs.

## Results

### Synchronization of sexual and asexual embryos

**Figure 1:**
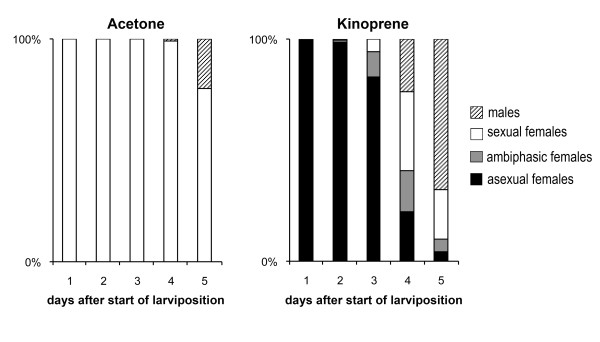
**Effect of prenatal application of kinoprene on the type of embryonic ovaries**. Acetone or kinoprene dissolved in acetone were topically applied to sexuparae individuals, 24 h after the fourth instar molt. Progeny batches were collected after initiation of larviposition, for five days every 24 h. The reproductive phenotypes of the progeny were determined and the percentage of each morph (asexual females, ambiphasic females that contain a mixture of eggs and embryos, sexual females and males) is indicated. In control conditions (acetone), sexuparae sequentially produced sexual females and males: only sexual females were produced during the first three days after the onset of larviposition; males appeared the fourth day. Sexuparae treated with kinoprene produced exclusively parthenogenetic progeny the first day after the onset of larviposition. On day two, almost all the progeny were parthenogenetic. On day three, the majority of the progeny were still parthenogenetic. On days four and five, sexual morphs represented the majority of the progeny.

The development of aphid embryos under long and short photoperiods is not synchronous. However, comparing the embryonic transcriptomes produced during sexual and asexual oogenesis requires synchronized embryo development. Thus, we used kinoprene (a JH analogue) to synchronize the development of sexual and asexual embryos under otherwise identical environmental conditions. Topical applications of kinoprene induces sexuparae reared under short photoperiods to produce asexual embryos instead of sexual embryos [11]. Compared to acetone-treatment (control), kinoprene treatment induced complete reversion of sexual to asexual reproduction in all larvae laid during the first two days after the onset of larviposition (Figure 1). Subsequent transcriptome comparisons were thus restricted to the five most-developed embryos dissected from synchronized kinoprene- or acetone-treated sexuparae. These embryos corresponded to the progeny laid on the first day.

### Differentially expressed transcripts

Synchronized asexual (kinoprene-treated) and sexual (acetone-treated) embryos corresponding to embryonic developmental stages 18, 19 and 20, were collected from sexuparae 24 h, 48 h and 72 h after treatment, respectively. These are the final three developmental stages in aphid embryogenesis and correspond to eye differentiation (stage 18), muscle formation (stage 19) and the mature embryo (stage 20) [8]. We compared the transcriptomes of these embryos using microarrays. Pairwise correlations between the five biological replicates were high (r > 0.88), indicating good reproducibility. We compiled a list of 33 transcripts that were statistically differentially expressed: none of these transcripts were differentially regulated at developmental stage 18. Only one transcript (*orb*) was more abundant in asexual embryos than in sexual embryos at developmental stage 19, whereas 33 transcripts (including *orb*) were differentially-regulated at developmental stage 20, including *orb*. At this final stage, four transcripts were more abundant in sexual embryos than in asexual embryos, with fold-change (FC) values between 1.49 and 2.86. Twenty-nine transcripts were more abundant in asexual embryos than in sexual embryos (FC values between 1.69 and 5.70) (Table 1).

**Table 1:** Description of the 33 differentially-expressed transcripts in sexual and asexual embryos

Functional group	Gene identity	Similarity	Commentaries	Fold change
Oogenesis (7)	*ACYPI009492-RA*	*nudel*	oocyte dorsal/ventral axis formation, egg activation	3.86
	*ACYPI56634-RA_0*	*orb*	oocyte axis specification, mRNA polyadenylation, germ cell development	4.67
	*ACYPI008811-RA*	*bicaudal-C*	mitosis, oogenesis, ovarian follicle cell migration	4.69
	*ACYPI31336-RA*	*kelch*	female germline ring canal formation, karyosome formation, oogenesis	1.81
	*Ap-nanos1*	*nanos*	female meiosis chromosome segregation, germ cell development, oogenesis	3.69
	*ACYPI23247-RA*	*putative kelch-like*	female germline ring canal formation, karyosome formation, oogenesis	1.69
	*ACYPI46386-RA*	*putative kelch-like repeat*	female germline ring canal formation, karyosome formation, oogenesis	-1.49
Post-transcriptionnal	*ACYPI00753-RA*	*lodestar*	termination of RNA polymerase II transcription, female meiosis	3.39
regulation (5)	*ACYPI003103-RA*	*Gld2*	RNA polyadenylation, histone mRNA catabolic process, mRNA processing	4.03
	*ACYPI50630-RA*	*Gle1*	poly(A)+ mRNA export from nucleus	2.46
	*ACYPI001842-RA*	*Pop2*	RNA metabolic process, nuclear-transcribed mRNA poly(A) tail shortening	2.21
	*ACYPI49482-RA*	*cbp20*	gene silencing by miRNA, production of siRNA involved in RNA interference	2.11
Epigenetic	ACYPI002182-RA	histone H2B.3	chromatin assembly or disassembly, nucleosome assembly	3.60
regulation (4)	ACYPI005639-RA	histone H1	chromatin assembly or disassembly, nucleosome assembly	2.85
	ACYPI28709-RA	*Suv4-20H1*	histone methylation, regulation of gene expression, epigenetic	1.75
	ACYPI007975-RA	*uhrf1*	DNA repair, cell cycle, methylated histone residues binding	2.48
Cell cycle (3)	*ACYPI006224-RA*	*cyclin J*	mitotic cell cycle, embryonic	5.70
	*ACYPI007770-RA*	*clasp1*	cell division, exit from mitosis, condensed chromosome kinetochore	1.48
	*ACYPI009671-RA*	*Drp-1*	cytokinesis, intracellular distribution of mitochondria	1.79
Other (4)	ACYPI48893-RA	*Arl6ip1*	cotranslational protein targeting to membrane	4.25
	*ACYPI48336-RA*	*Lsd-1*	lipid particle organization, regulation of lipid storage	-2.86
	*ACYPI34416-RA*	*pol protein*	RNA dependant DNA Polymerase/Reverse Transcriptase	-1.92
	*ACYPI010082-RA*	*Six4*	fat body development, gonad development, mesoderm development	1.56
No similarity (10)	ACYPI50301-RA	no similarity		3.92
	ACYPI39347-RA	hypothetical protein		2.70
	ACYPI38914-RA	predicted protein		2.04
	ACYPI54656-RA	hypothetical protein		2.12
	ACYPI25970-RA	hypothetical protein		3.86
	*ACYPI010052-RA*	hypothetical protein		3.78
	*ACYPI007465-RA*	hypothetical protein		2.53
	ACYPI25088-RA	hypothetical protein		2.20
	*ACYPI39770-RA*	no similarity		2.88
	ACYPI005121-RA	hypothetical protein		-1.78

Positive fold-change values indicate transcripts that were more abundant in asexual embryos. Negative fold-change values indicate transcripts that were more abundant in sexual embryos.

Based on gene annotation in other organisms, 4 functional groups were defined (Table 1). Seven transcripts were related to oogenesis (e.g. *nanos-1, orb, kelch*); five transcripts were related to post-transcriptional regulation, such as mRNA polyadenylation (e.g. *gld2*) or mRNA silencing via RNA interference (*cbp20*); four transcripts were related to epigenetic regulation, such as histone modifications (*suv4-20, uhrf1*) or histone variants (*H2B.3, H1*); and three transcripts were assigned to cell cycle regulation (e.g. *cyclin J, clasp1*). Fourteen transcripts were not assigned to any functional category: among them, ten showed no similarity with characterized transcripts.

Our results displayed multiple genes related to oogesis (7/33). This oogenesis signature suggests that our experimental design, using artificially-induced asexual aphids, successfully targeted genes defining asexual or sexual oogenesis within the germaria or the first ovulated oocytes of the developing embryo.

### Localization of regulated transcripts in sexual and asexual ovaries

We compared the localization of these differentially-expressed transcripts in the ovaries of untreated asexual and sexual aphids using whole-mount *in situ *hybridization (WISH), a technique that is not suitable for transcript quantification. Specific antisense riboprobes were produced for 25 transcripts: five produced no signal (Additional file 1), four showed ubiquitous distribution (Additional file 2) and 16 transcripts were specifically expressed in germaria (containing germ and nurse cells) and/or in oocytes. These results on untreated aphids were consistent with the microarray data from acetone- or kinoprene-treated aphids: most of the candidate genes are expressed in the ovarioles. These 16 transcripts were grouped based on their patterns of expression in the two types of ovaries.

**Figure 2:**
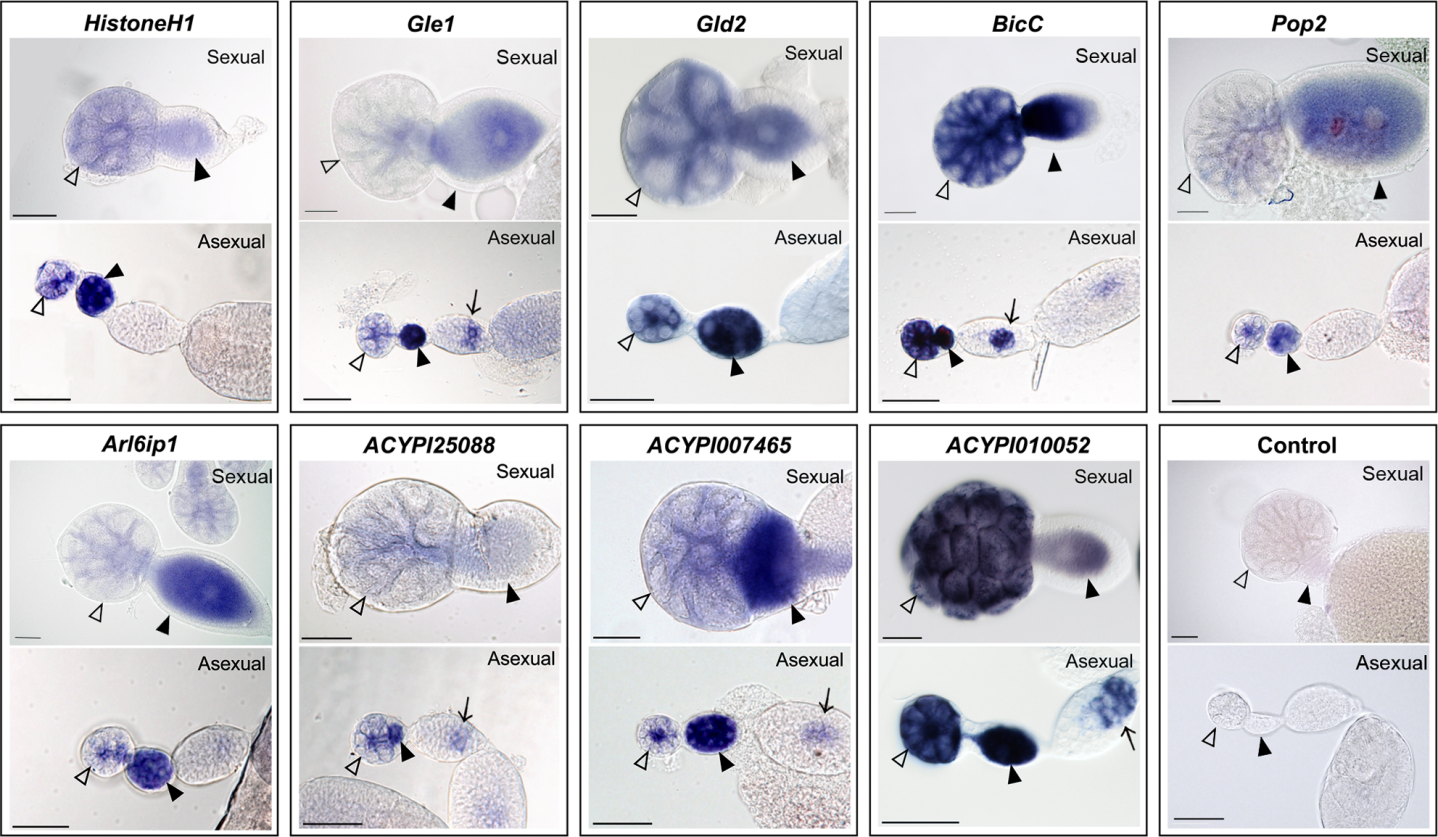
**Transcripts with identical localizations in both sexual and asexual ovaries**. *Histone H1, gle1, gld2, bicC, pop2, arl6ip1, ACYPI25088, ACYPI007465 *and *ACYPI10052 *transcripts were detected in germaria (hollow arrowheads) and oocytes (black arrowheads) of both sexual and parthenogenetic ovaries. Sense riboprobes were used as negative controls. For *gle1, bicC, ACYPI25088, ACYPI007465 *and *ACYPI010052*, a residual signal was observed in the developing embryo germ band (arrows). Bar scale: 50 μm.

The first group included nine transcripts (*bicC, histoneH1, gle1, gld2, pop2, arl6ip1, ACYPI007465, ACYPI010052 *and *ACYPI25088*) that showed the same localization in sexual and asexual ovaries (Figure 2) despite microarray results that indicated higher levels of expression in asexual embryos. For five transcripts (*gle1, bicC, ACYPI25088, ACYPI007465 *and *ACYPI010052*), residual signals were observed in the developing embryo germ band (Figure 2).

**Figure 3:**
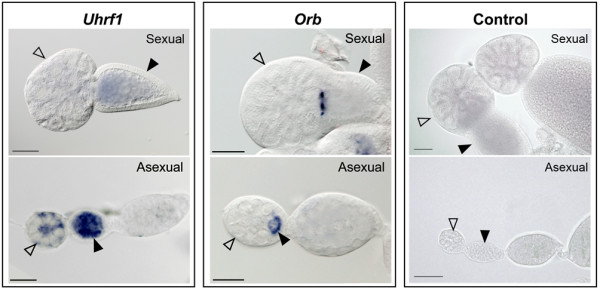
**Transcripts with different localizations in sexual and asexual ovaries**. *Uhrf1 *transcripts were detected in germaria (hollow arrowheads) and oocytes (black arrowheads) of asexual ovaries whereas a weak signal was detected in sexual oocytes (black arrowheads). *Orb *transcripts were detected in the basal parts of germaria in asexual and sexual ovaries (hollow arrowheads) with distinct localizations. In asexual germaria, these cells correspond to oocytes, whereas the cellular structures containing *orb *transcripts in sexual ovaries are undetermined. Sense riboprobes were used as negative controls. Bar scale: 50 μm.

**Figure 4:**
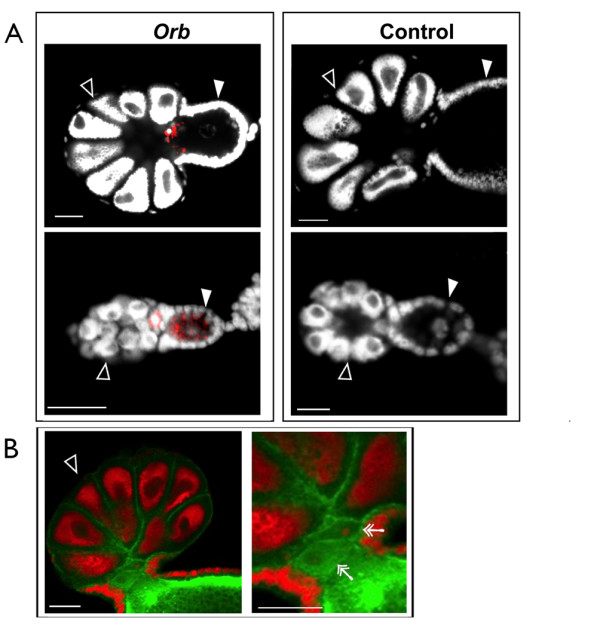
**Detection of *orb *transcripts in sexual and asexual ovaries by fluorescent WISH**. (A) *orb *riboprobes were detected with an antibody coupled to alexin fluorochrome (red) and DNA was stained with DAPI (white). In sexual ovaries (upper pictures), *orb *transcripts were detected at the basal part of the germaria (hollow arrowheads). In asexual ovaries (lower pictures), *orb *transcripts were detected in oocytes (white arrowheads) before ovulation (stage 0), and after ovulation (stage 1). (B) Actin filaments and DNA in sexual ovaries were stained with phalloidin (green) and propidium iodide (red), respectively. Cells at the basal part of the sexual germaria possibly correspond to presumptive sexual oocytes (double arrows). *Orb *sense riboprobes were used as negative controls. Bar scale: 30 μm.

The second group included two transcripts (*uhrf1 *and *orb*) that exhibited different localizations in the two types of ovaries. *Uhrf1 *signal was detected in germaria and oocytes in asexual ovaries, and a weak signal was detected in sexual oocytes (Figure 3). *orb *was detected in oocytes prior to ovulation (stage 0), in asexual germaria, and in unknown cellular structures located at the junction between sexual germaria and oocytes. Fluorescent detection of *orb *riboprobes was used together with fluorescent staining of actin filaments and DNA in order to define the intracellular localization of *orb *transcripts (Figure 4). In asexual ovaries, *orb *signals could be seen during early oogenesis, in stage 0 (oocyte formation) and in stage 1 (separation of the oocyte from the germarium) (Figure 4A). In sexual ovaries, the transcripts were detected only before ovulation at the junctions between the sexual germaria and the oocytes (Figure 4A). According to actin staining, a group of cells is localized at the junctions between sexual germaria and oocytes, these cells may correspond to the presumptive oocytes (Figure 4B). Thus, in asexual germaria, *orb *transcripts were detected in oocytes but not in presumptive oocytes; whereas in sexual ovaries, these transcripts were detectable only in cells that probably corresponded to presumptive oocytes but were not detectable in mature oocytes.

**Figure 5:**
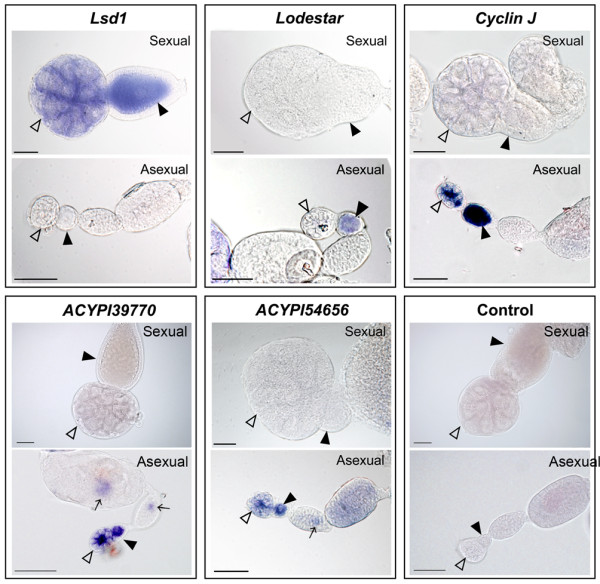
**Transcripts detected specifically in one type of ovary**. *Lsd1 *transcripts were detected specifically in the germaria (hollow arrowheads) and haploid oocytes (black arrowheads) of sexual ovaries. *lodestar, cyclin J, ACYPI39770, ACYPI54656 *transcripts were detected specifically in asexual ovaries. *Lodestar *signal was detected specifically in asexual oocytes (black arrowheads). *Cyclin J, ACYPI54656 *and *ACYPI39770 *signals were detected in germaria (hollow arrowheads) and in oocytes (black arrowheads) of asexual ovaries. *ACYPI54656 *and *ACYPI39770 *showed a residual signal in the germ bands (arrows) of developing embryos. Sense riboprobes were used as negative controls. Bar scale: 50 μm.

The third group included five transcripts (*lsd1, lodestar, cyclin J, ACYPI39770 *and *ACYPI54656*) that could be localized only in one type of ovary (Figure 5). These specific expression patterns were confirmed by microarray expression data for *lsd1, lodestar, cyclinJ *and *ACYPI39770*. The *lsd1 *riboprobe showed a strong specific signal in the germaria and oocytes of sexual ovaries. In contrast, a specific signal for *lodestar, cyclin J, ACYPI39770 *and *ACYPI54656 *was detected only in asexual ovaries (Figure 5). *lodestar *transcripts were restricted to the diploid oocyte after separation from the germarium (stage 1). *cyclin J, ACYPI39770 *and *ACYPI54656 *transcripts were detected in asexual germaria and oocytes (Figure 5). In addition, *ACYPI54656 *and *ACYPI39770 *exhibited a residual signal in the germ bands of developing embryos (Figure 5).

## Discussion

By coupling quantitative transcriptomic and qualitative WISH approaches, we identified 33 transcripts associated with the onset of divergent transcripts expression leading to the establishment of the sexual or asexual phenotype in aphids. The involvement of these candidate genes in determining the developmental direction of oogenesis is supported by: i) a strong signature of oogenesis among the regulated transcripts (7/33); and ii) the oocyte- and/or germarial-specific localizations of 16 of the transcripts. These data illustrate both shared and unique regulatory patterns of transcript expression for the two modes of reproduction.

First, we identified transcript expression profiles shared by sexual and asexual oogenesis. These transcripts are expressed in sexual and asexual germaria and/or oocytes but display differences in their expression levels and/or in their localization. In various organisms, *gld2, bicC, pop2 *and *orb *are all involved in regulating the poly(A) tail length of maternal mRNAs. This process is a common mechanism of translational control essential for progression of meiosis and axis patterning during oocyte maturation [12-14]. The regulation of poly(A) tail length of maternal mRNAs results from a balance between concomitant deadenylation (translation repression) and polyadenylation (translation activation) [15]. In *Drosophila melanogaster, gld2, bicC *and *orb *have been described as part of the cytoplasmic polyadenylation complex that polyadenylates mRNAs and activates their translation [13,16]. Gld2-dependent mRNA polyadenylation and translational activation have been shown to be essential for oogenic meiosis [13]. On the other hand, mRNA deadenylation by the major deadenylation complex in *Drosophila*, CCR4-NOT, which includes the deadenylases CCR4, POP2, and four NOT proteins [17], promotes negative regulation of target mRNAs. Deadenylation of specific maternal mRNAs could be involved in the precise temporal and spatial mRNA localization necessary for axial patterning during oogenesis [12]. Expression of *Api-gld2, Api-bicC, Api-pop2 *and *Api-orb *in sexual and asexual germaria and/or in oocytes suggests that the balance between cytoplasmic polyadenylation and deadenylation in oocytes acts in the maturation of haploid as well as diploid oocytes in the pea aphid as it has been shown for other sexual organisms. Moreover, expression of the oocyte-specific linkers *histoneH1 *and *uhrf1 *in asexual and sexual oocytes suggests that epigenetic regulation is involved in pea aphid oogenesis. The linker histone H1 interacts with DNA to set-up and maintain the organization of the chromatin between the nucleosomes. Histone H1 proteins form a complex family of related proteins that have distinct species, tissue and developmental specificities and are involved in the epigenetic control of gene expression. The replacement of a somatic linker *H1 *by an oocyte specific one (*H1oo*) during oogenesis has been described in a wide range of species (reviewed in [18,19]) and may promote a loosening in chromatin structure that appears to correlate with the initiation of meiosis [19]. The presence of *Api-histoneH1 *in germaria and oocytes suggests that this oocyte-specific variant of H1 might be involved in both sexual and asexual oogenesis in aphid. *uhrf1 *encodes an ubiquitin-like protein that is involved in two epigenetic silencing pathways through its regulation of DNA and histone methylation [20,21]. Although, *uhrf1 *has not been implicated in any oogenesis process to date, our results suggest a role for this protein in asexual and sexual oogenesis in aphids. Altogether, our results suggest that epigenetic regulatory mechanisms and the balance between cytoplasmic mRNA polyadenylation and deadenylation may have roles in regulating haploid and diploid oocyte maturation. Moreover, as *orb *transcript is the earliest to be differentially expressed during the developmental time course, we propose that Orb might play a major role in determining oocyte fate.

Second, we identified one gene, *lsd1*, that is specifically expressed in pea aphid sexual germaria and oocytes. The *lsd1 *transcript was not significantly detected either in asexual embryos according to microarray data (Additional file 3) or in asexual ovaries according to WISH. *lsd1 *encodes a protein involved in the regulation of lipid droplet storage in eukaryotic cells [22]. Lipid droplets, like mature yolk bodies, are the major energy storage for oocytes and developing embryos in *Drosophila *[23]. Our results suggest that *lsd1 *is required only for sexual oogenesis during which it may be involved in yolk accumulation. The absence of *lsd1 *transcripts in asexual embryos is consistent with the absence of yolk in parthenogenetic oocytes [8]. Thus, identification of *lsd1 *expression as a specific feature of sexual oogenesis validates the accuracy of our approach. Despite the existence of genes known to be involved in meiotic recombination in the pea aphid genome [24], we identified no such genes as specific markers of sexual oocytes. However, germline expression of meiotic genes such as *spo11 *was shown to be similar in sexual and asexual aphids. Expression of this gene seems to be modulated between asexual and sexual aphids by alternative splicing [25].

Finally, we identified three transcripts that were specific to asexual oogenesis. No significant transcription signal has been observed for these genes either in sexual embryos using microarrays (Additional file 3) or in sexual ovaries using WISH. That the polypeptide encoded by *ACYPI39770 *does not bear any similarity to a protein or protein domain of any other organism, suggests that *ACYPI39770 *is either an orphan gene or a gene with an unusual accelerated evolution rate. In *Drosophila, cyclin J *and *lodestar *are involved in sexual oogenesis and early embryogenesis [26,27]. In aphids, their expression profiles suggest that they may haveroles in asexual oogenesis only. The absence of *lodestar *and *cyclin J *transcripts from aphid sexual oocytes and embryos was particularly unexpected. Three *lodestar *paralogs were identified within the pea aphid genome (*ACYPI000753, ACYPI000590 *and *ACYPI006971*). Although the three copies were represented on the microarray by copy-specific oligonucleotides, only *ACYPI000753 *was differentially expressed in sexual and asexual embryos. Localization of *ACYPI000753 *transcripts by *in situ *hybridization showed that its expression was specific to asexual oocytes. However, although the sequence of the riboprobe used to locate *ACYPI000753 *transcripts was absent from the *ACYPI006971 *sequence, it shared 87% nucleotide identity with the unregulated paralog *ACYPI000590*. Therefore, cross hybridization between the *ACYPI000753 *riboprobe and *ACYPI000590 *transcripts cannot be excluded. Nevertheless, we detected no *ACYPI000753 *expression in sexual oogenesis. This unexpected result might reflect the functional specialization of at least one of the *lodestar *paralogs in asexual oogenesis.

Among the 16 transcripts found to be specifically localized in germaria and/or oocytes *, lsd1 *was the only one for which the expression level was found to be higher in sexual ovaries than in asexual ovaries according to the microarray results. This disproportion is consistent with the striking over-representation of transcripts that were upregulated in asexual embryos (29/33), as evidenced by the microarray analysis. Sexual oogenesis and asexual oogenesis display major inherent differences. Within embryos, the sexual oocyte nucleus remains blocked in meiosis prophase 1 whereas the asexual oocyte undergoes a modified but complete meiotic division immediately after ovulation [7]. This modified meiosis involves specific changes within the oocyte; self-organized asters are formed, that then recruit centriole precursors and pericentriolar material [28]. This process may be accompanied by the activation of microtubule stabilization factors or by the inactivation of microtubule destabilization factors specific to asexual oogenesis [28]. The predominance of genes that are upregulated in asexual embryos may be partially explained by the production of molecular components that are specifically required for asexual oogenesis but not for sexual oogenesis.

## Conclusions

In this study, we identified a number of putative embryonic target genes of the signaling cascade initiated by photoperiodic cues and transduced by JH. These genes are likely to be among those that direct sexual or asexual differentiation of the developing embryos. Our analysis of their expression patterns represents the first time the molecular bases of aphid sexual and asexual oogenesis have been addressed. Altogether, our results showed that aphid asexual oogenesis requires the same genes as sexual oogenesis but that these two distinctive differentiation processes display specific gene expression programs.

## Methods

### Aphid rearing, dissection and extraction

Clonal descendants from the sequenced *Acyrthosiphon pisum *LSR1 clone were reared on *Vicia fabae *at 18°C and at low density (5 individuals per plant) to prevent the production of winged morphs. Parthenogenesis was maintained under long photoperiod (16 h). In our conditions, the shortening of the photoperiod (12 h) requires three generations to induce the production of sexual morphs. The first asexual generation was reared under long photoperiod and transferred to short photoperiod (12 h) after the third instar moult. Adult asexual virginoparae females produced the second asexual generation, named sexuparae. Adult sexuparae, reared in short photoperiods, produced the third generation of sexual females oviparous, males, and asexual viviparous females.

### Kinoprene application

Aphid sexuparae were synchronised at the fourth instar moult, during a 6 hour window. 24 h after, 400 ng of kinoprene (Sigma Aldrich) diluted in 50 μL of acetone were ectopically applied on sexuparae abdomens of 100 females. The progeny of treated aphids was collected daily for 5 days. When they reached adulthood, collected aphids were dissected and their reproductive type was determined according to the type of ovaries they contained within their abdomen: sexual ovaries that are only made of haploid eggs, ambiphasic ovaries that contain a mixture of eggs and embryos, and asexual ovaries that are only constituted of embryos at different stages of development. Ectopic applications of 50 μL of acetone were performed as a negative control.

### Sampling, RNA isolation and microarray hybridization

Synchronized sexuparae were randomly divided into 2 batches and treated with kinoprene or acetone (as a control) 24 h after fourth instar moult (Additional file 4). 25 treated sexuparae were collected 24, 48, and 72 hours after kinoprene or acetone application. For each condition, the 5 most developed embryos were isolated from each of the 25 treated sexuparae by dissection, pooled together, frozen into liquid nitrogen and stored at -80°C. These most developed embryos within treated sexuparae collected 24, 48, and 72 hours after treatment correspond to the developemental stage 18, 19 or 20 respectively. This procedure was repeated 5 times to generate as much independent biological replicates. Total RNAs were isolated from each sample by using the RNeasy Mini kit (Qiagen) according to manufacturer's instructions. RNA quality was checked on Bioanalyser (Agilent) and quantified on Nanodrop (Thermo scientific). For each sample, 20 μg of total RNAs were sent to the NimbleGen expression array platform (Roche). Double stranded cDNA synthesis and Cy3 end-labelling were performed by NimbleGen.

### Microarrays design and analysis

Custom microarrays were constructed on NimbleGen (Roche) 385K 4-plex (4 × 72,000 probes). 24,011 transcripts were represented by 3 60-mers oligonucleotides probes (MIAMExpress, http://www.ebi.ac.uk/miamexpress/, microarray: INRA-BF2I_A.pisum_Nimblegen-ACYPI_4x72k_v1; Array express accession: A-MEXP-1999). Hybridization and scanning were performed by NimbleGen, providing the final raw data file (experiment: Aphid_embryo, ArrayExpress accession: E-MEXP-3481). The limma 2.16 package [29] in R 2.9.2 [30] was used for statistical analyses. For each transcript the average of the 3 probe signals was considered. Median normalization between microarrays was performed. A design matrix incorporating effects of treatment K (kinoprene) or A (acetone) and developing stage (18, 19, 20) was constructed. Variance was adjusted for Bayesian fitting of the model. Differential expression was determined with a two-way ANOVA considering the effect of time and treatment between 3 contrasts: 'A18 *versus *K18', 'A19 *versus *K19' and 'A20 *versus *K20'. Significance of differential expression was assigned with a 10% false discovery rate (FDR) [31]. Annotation of differentially expressed transcripts was curated using pea aphid RNA-seq data (published in SRA of NCBI), the software CAP3 [32] and Blastx on NCBI non-redundant protein sequence data bank. Functional annotations were performed using the Gene Ontology (http://www.geneontology.org/).

### Riboprobe synthesis for *in situ *hybridization

Templates for synthesis of riboprobes were obtained from full-length cDNAs collection (*ACYPI003103, ACYPI010052 *[33]) or amplified by RT-PCR and cloned (Additional file 5). Total RNAs were extracted from parthenogenetic virginoparae females with RNeasy plant kit (Qiagen). DNA contaminations were removed by a treatment with RQ1 RNase-free DNAse (Promega). First strand cDNAs were produced from 1 μg of total RNAs by using random primer 9 (New England BioLabs) and SuperScript^® ^III Reverse Transcriptase (Invitrogen) following the supplier's instructions. cDNAs were used as a matrix for PCR amplification with specific primers (Additional file 5). Amplified fragments were cloned into the StrataClone PCR Cloning Vector pSC-A-amp/kan (StrataClone) or pENTR Directional TOPO (Invitrogen) and sequenced (Genoscreen). Linear PCR products were amplified from cloned sequence with universal primers M13 and used as a matrix for synthesis of sense and antisense riboprobes by using digoxigenin-labelled dNTPs and the appropriate RNA polymerase (T3/T7/SP6) supplied in the DIG RNA labelling kit (Roche). Remaining DNA was removed with RQ1 RNase-free DNAse treatment (Promega) and riboprobes were purified with the RNeasy mini kit (Qiagen). Riboprobe quality and quantity were checked on Nanodrop (Thermo scientific).

### Whole mount *in situ *hybridization and microscopy

Whole mount *in situ *hybridization was performed on ovaries of sexual or asexual nymphs. Ovaries were dissected and fixed in 4% paraformaldehyde in 1× PBS at room temperature (RT) for 30 min. Ovaries were washed in 50% methanol for 30 min, then dehydrated and stored in methanol at -20°C. They were rehydrated in graded methanol/PTw (1× PBS, 0.2% Tween-20) solutions (70%; 50%; 30%, 10 min each), post fixated in 4% paraformaldehyde in 1× PBS for 20 min and washed 3 times for 5 min in PTw. Ovaries were washed for 45 min in 1% SDS, 0.5% Tween-20, 50 mM Tris-HCl (pH 7,5), 1 mM EDTA (pH 8), 150 mM NaCl and 5 times for 5 min with PTw. Pre-hybridization was performed 10 min in hyb-wash solution (5× SSC, 50% formamide, 0.1% Tween-20) and 1 hour at 65°C in hyb solution (0.3% SDS, 5× SSC, 50% formamide, 100 μg/mL heparin, 0.1% Tween-20, 100 μg/mL yeast RNA, 10 mM DTT). Hybridization was performed overnight at 65°C with 500 ng/mL of denatured sense or antisense RNA probe diluted in hyb solution. Nonspecific hybridizations were washed off twice at 65°C in hyb-wash solution for 30 min, 3 times in 50% hyb-wash solution/PTw 30 min and 3 times in PTw for 10 min at RT. Ovaries were incubated in blocking solution (0.2% BSA in PTw) for 5 min and 1 h at RT, and bound digoxigenin labelled probes were detected overnight at 4°C with anti-DIG-alkaline phosphatase (AP) Fab fragments (Roche) diluted 1:2000 in blocking solution. Ovaries were washed 4 times 20 min and twice for 1 h in blocking solution at RT. After 3 washes in AP reaction buffer (100 mM Tris (pH 9,5), 100 mM NaCl, 5 mM MgCl_2_, 0.2% Tween-20), signal was revealed with 4 μl NitroBlue Tetrazolium/5-Bromo-4-Chloro-3-Indolyl Phosphate (NBT/BCIP) Stock Solution (Roche)/ml AP reaction buffer. Finally, ovaries were rinsed at least 3 times for 5 min in PTw, dehydrated 5 min in methanol, and rinsed twice again 5 min in PTw, before mounting in 70% glycerol in PBS. Samples were photographed with a microscope Nikon 90i connected to a Nikon type DS-Ri1 camera or with Olympus BX61 connected to Nikon DS-Fi1 camera.

For the fluorescence protocol, the same procedure was followed until hybridization step. Then TSA™ Biotin System (Perkin Elmer, Waltham, USA) was used for detection as follows. After overnight hybridization of the probes, nonspecific probes were washed off twice at 65°C in hyb-wash solution for 30 min, once in 50% hyb-wash solution/TNT buffer (0.1 M Tris-HCl (pH 7.5), 0.15 M NaCl, 0.05% Tween-20) for 30 min at RT, and 3 times in TNT buffer for 10 min at RT. Ovaries were blocked with the TSA™ blocking reagent, for 30 min at RT. Bound digoxigenin labelled probes were detected with HRP conjugated anti-DIG (Boehringer-Mannheim) (1:250) diluted in TNT buffer at 4°C for overnight incubation. Ovaries were washed four times in TNT buffer for 15 min. The TSA™ Biotin System Amplification was used by incubation 20 min into a diluted solution of the Biotinyl Tyramide (Amplification Reagent) in 1× Amplification Diluent (1:50). HRP catalyzes the formation of TSA free radicals, which form covalent bounds to tyrosine residues proximal to HRP. They were then washed 3 times 15 min in TNT buffer, and 4 times 20 min in PTw. Ovaries were incubated 2 h into Alexa 594-conjugated Streptavidin (Invitrogen) diluted in TNT buffer (1:500) and rinsed into PTw before performing a nuclear stain with TO-PRO3^® ^(1:1000 in PTw) for 30 min. Finally, ovaries were washed 3 times in PTw before mounting. Images were acquired and processed using an Olympus FV1000 confocal microscope.

### Phalloidin and propidium iodide staining

Oviparous ovaries were dissected and fixed as previously described. Samples were washed 3 times in PTw, followed by PTw for 30 min and 2% Normal Goat Serum (NGS)/BPTw for 60 min. Samples were then incubated overnight at 4°C with Alexa Fluor 488 phalloidin (Invitrogen) at a concentration of 1:200 to visualize F-actin. The next day samples were washed 4 times in PTw, and placed in 20 μg/mL RNaseA (QIAGEN)/PTw for 3 h at RT. Samples were washed twice in PTw, and incubated with 10 μg/mL propidium iodide (Invitrogen) in 20 μg/mL RNaseA/PTw for 60 min at RT to visualize DNA. Samples were washed 4 times in PTw and mounted in Vectashield (Vector Laboratories). Images were acquired and processed using an Olympus FV1000 confocal microscope.

## Authors' contributions

AG, SS, SJP and DT designed the research. AG and TH performed the experiments. AG, SS, SJP and DT analyzed data. AG, SJP and DT wrote the manuscript. All the authors read and approved the final manuscript.

## Author's information

AG, SJP and DT: INRA, UMR 1099 BiO3P, Biologie des Organismes et des Populations appliquée à la Protection des Plantes, 35653 Le Rheu cedex, France

SS and TH: Okazaki Institute for Integrative Bioscience, National Institute for Basic Biology, National Institutes of Natural Sciences, Higashiyama, Myodaiji, Okazaki, 444-8787, Japan

## Supplementary Material

Additional file 1 **Transcription of *nudel, drp1, clasp1, cbp1 *and *ACYPI005121 *in parthenogenetic ovaries**. *nudel, drp1, clasp1, cbp1 *and *ACYPI005121 *transcripts localizations were investigated by *in situ *hybridization on parthenogenetic ovaries including germaria (hollow arrowheads) and in oocytes (black arrowheads). Specific antisense riboprobes gave no signal for these transcripts. Sense riboprobes were used as negative controls. Bar scale: 50 μm. Click here for file

Additional file 2 **Transcription of *six4, suv4-20, kelch *and *ACYPI38914 *in parthenogenetic ovaries**. *six4, suv4-20, kelch *and *ACYPI38914 *transcripts localizations were investigated by *in situ *hybridization on parthenogenetic ovaries including germaria (hollow arrowheads) and in oocytes (black arrowheads). Specific antisense riboprobes showed ubiquitous distribution of these transcripts. Sense riboprobes were used as negative controls. Bar scale: 50 μm. Click here for file

Additional file 3 **Quantitative expression of *lsd1, lodestar, cyclin J *and *ACYPI39770 *in sexual and asexual embryos measured by microarrays**. Transcription levels of *lsd-1, lodestar, cyclin-J *and *ACYPI39770 *were measured by microarrays in sexual (full line) and asexual (dot line) embryos. Log 2 of expression value was provided for each developmental stage (18, 19 and 20). The grey area contained the values comprised between the first and third quartile calculated for the 24011 transcripts included in the microarray. Standard errors were measured for the 5 biological replicates. Click here for file

Additional file 4 **Experimental design to synchronize the development of pea aphid sexual and asexual embryos**. Sexuparae were synchronized at the fourth instar moult in a six hours window. Synchronous sexuparae were randomly separated into two batches; one treated with acetone (A) and one kinoprene (K) 24 h after fourth instar moult. Within each batch, sexuparae were collected 24 h, 48 h and 72 h after treatment, dissected and the 5 most developed embryos were collected for further RNA extraction. Click here for file

Additional file 5 **Primer sequences for cDNA amplification and riboprobe synthesis**. Specific PCR primers were designed for each of the regulated transcripts in order to amplify cDNA. Click here for file
