# Supplementary figures and images for: Sexual and asexual oogenesis require the expression of unique and shared sets of genes in the insect Acyrthosiphon pisum
Source: BMC Genomics. 2012 Feb 15;13:76. doi: 10.1186/1471-2164-13-76 (PMC3313892; doi:10.1186/1471-2164-13-76)

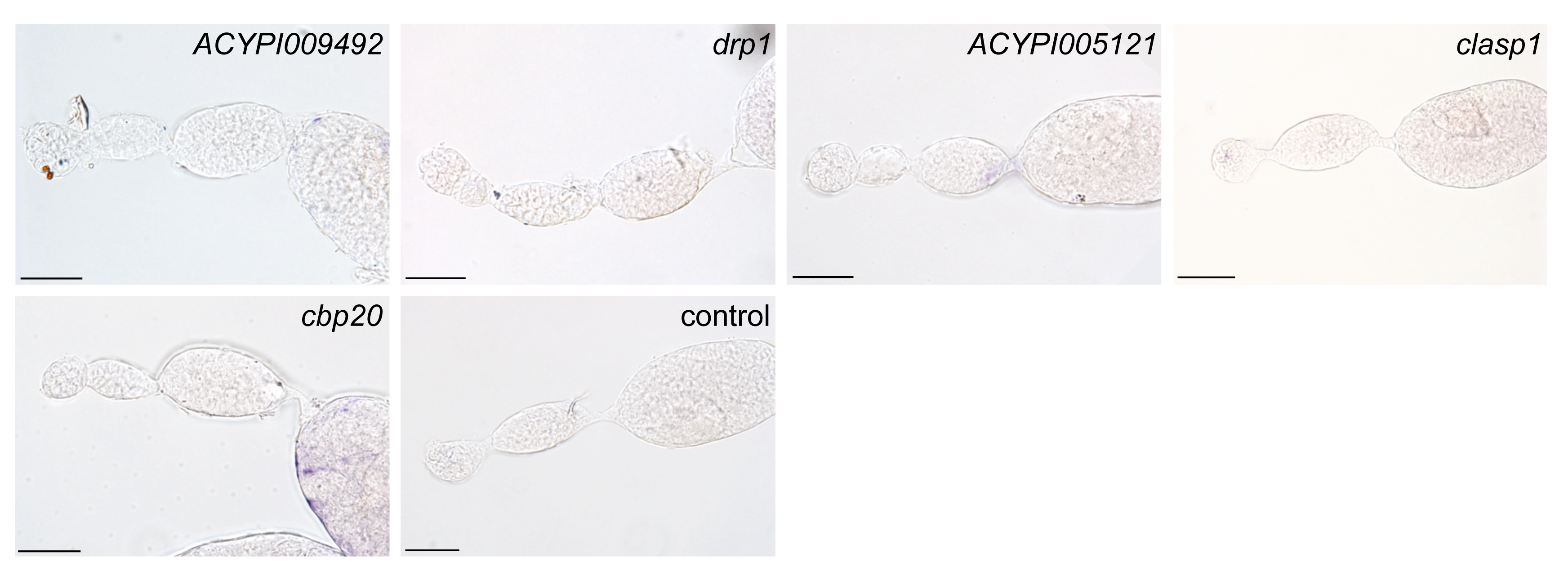

Supplement: Additional file 1 — Transcription of nudel, drp1, clasp1, cbp1 and ACYPI005121 in parthenogenetic ovaries. nudel, drp1, clasp1, cbp1 and ACYPI005121 transcripts localizations were investigated by in situ hybridization on parthenogenetic ovaries including germaria (hollow arrowheads) and in oocytes (black arrowheads). Specific antisense riboprobes gave no signal for these transcripts. Sense riboprobes were used as negative controls. Bar scale: 50 μm. [file 1471-2164-13-76-S1.TIFF]

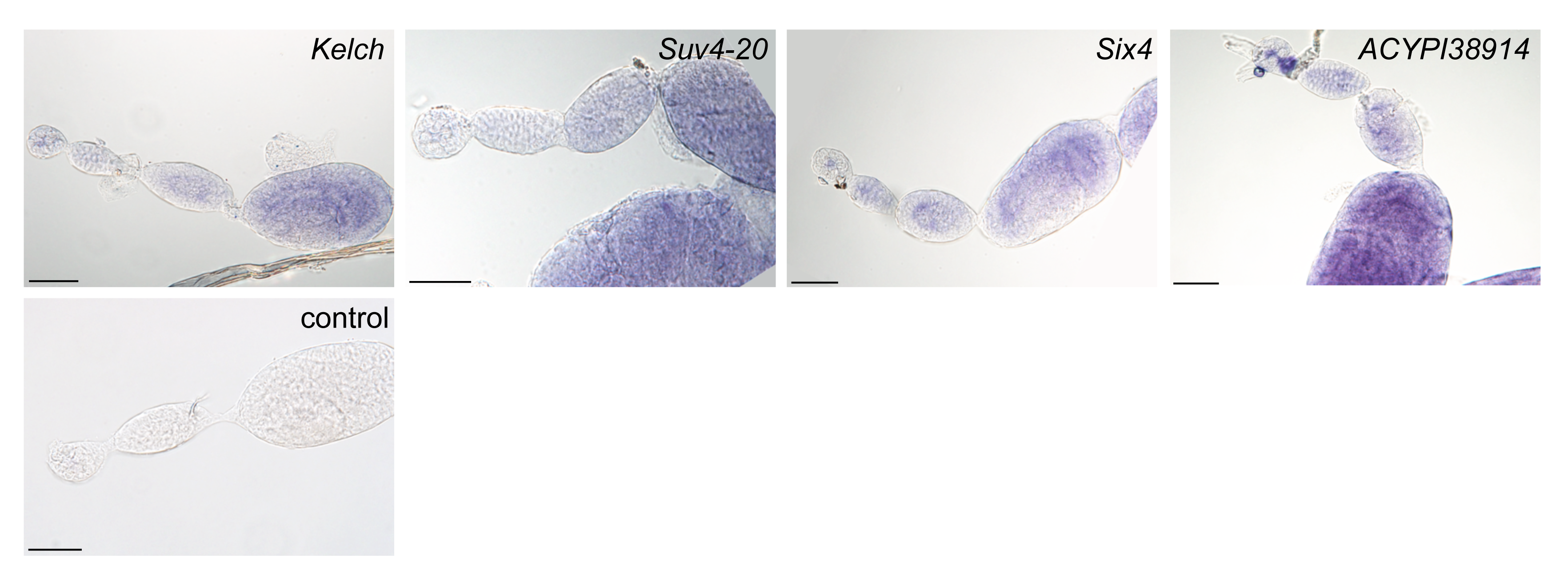

Supplement: Additional file 2 — Transcription of six4, suv4-20, kelch and ACYPI38914 in parthenogenetic ovaries. six4, suv4-20, kelch and ACYPI38914 transcripts localizations were investigated by in situ hybridization on parthenogenetic ovaries including germaria (hollow arrowheads) and in oocytes (black arrowheads). Specific antisense riboprobes showed ubiquitous distribution of these transcripts. Sense riboprobes were used as negative controls. Bar scale: 50 μm. [file 1471-2164-13-76-S2.TIFF]

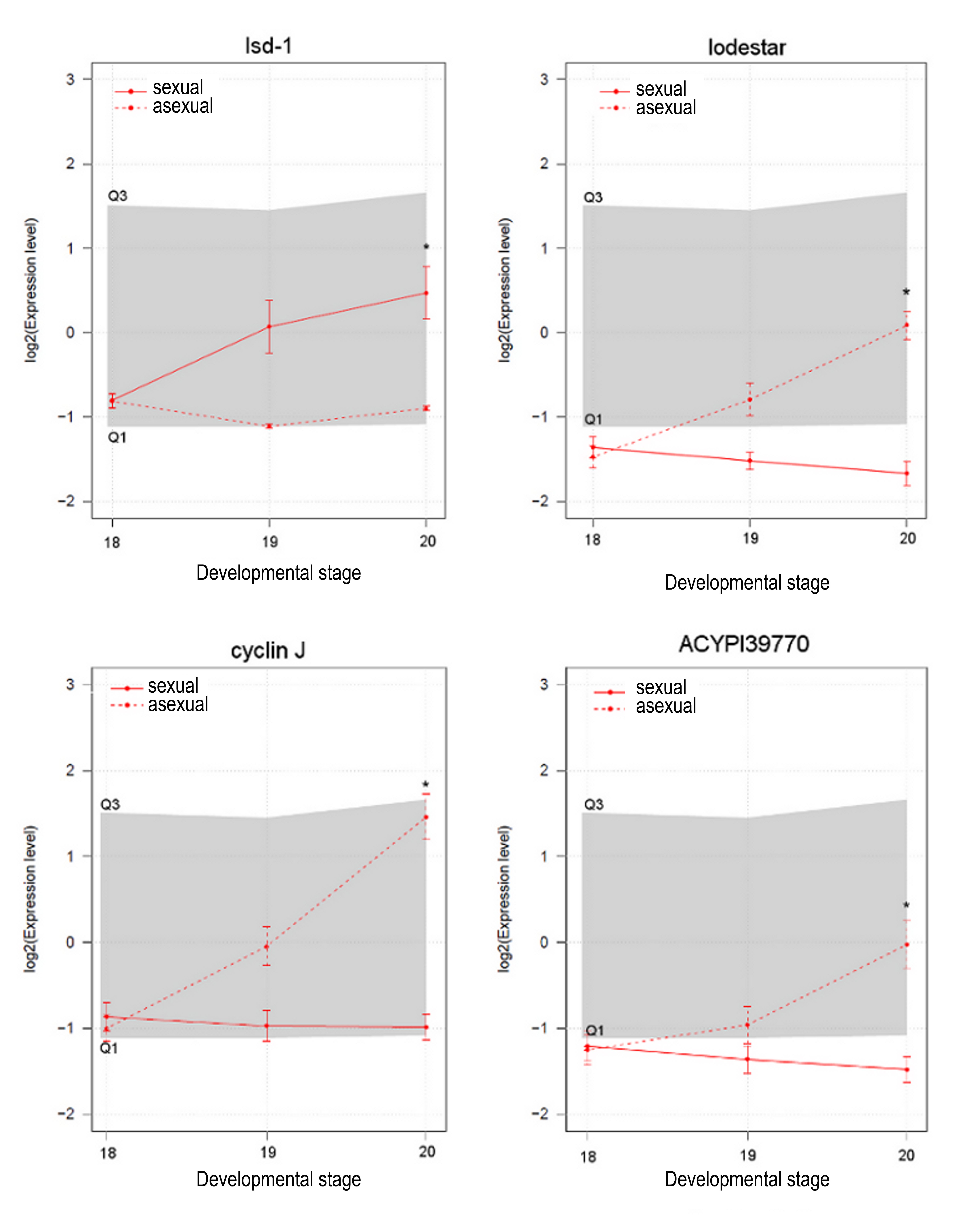

Supplement: Additional file 3 — Quantitative expression of lsd1, lodestar, cyclin J and ACYPI39770 in sexual and asexual embryos measured by microarrays. Transcription levels of lsd-1, lodestar, cyclin-J and ACYPI39770 were measured by microarrays in sexual (full line) and asexual (dot line) embryos. Log 2 of expression value was provided for each developmental stage (18, 19 and 20). The grey area contained the values comprised between the first and third quartile calculated for the 24011 transcripts included in the microarray. Standard errors were measured for the 5 biological replicates. [file 1471-2164-13-76-S3.JPEG]

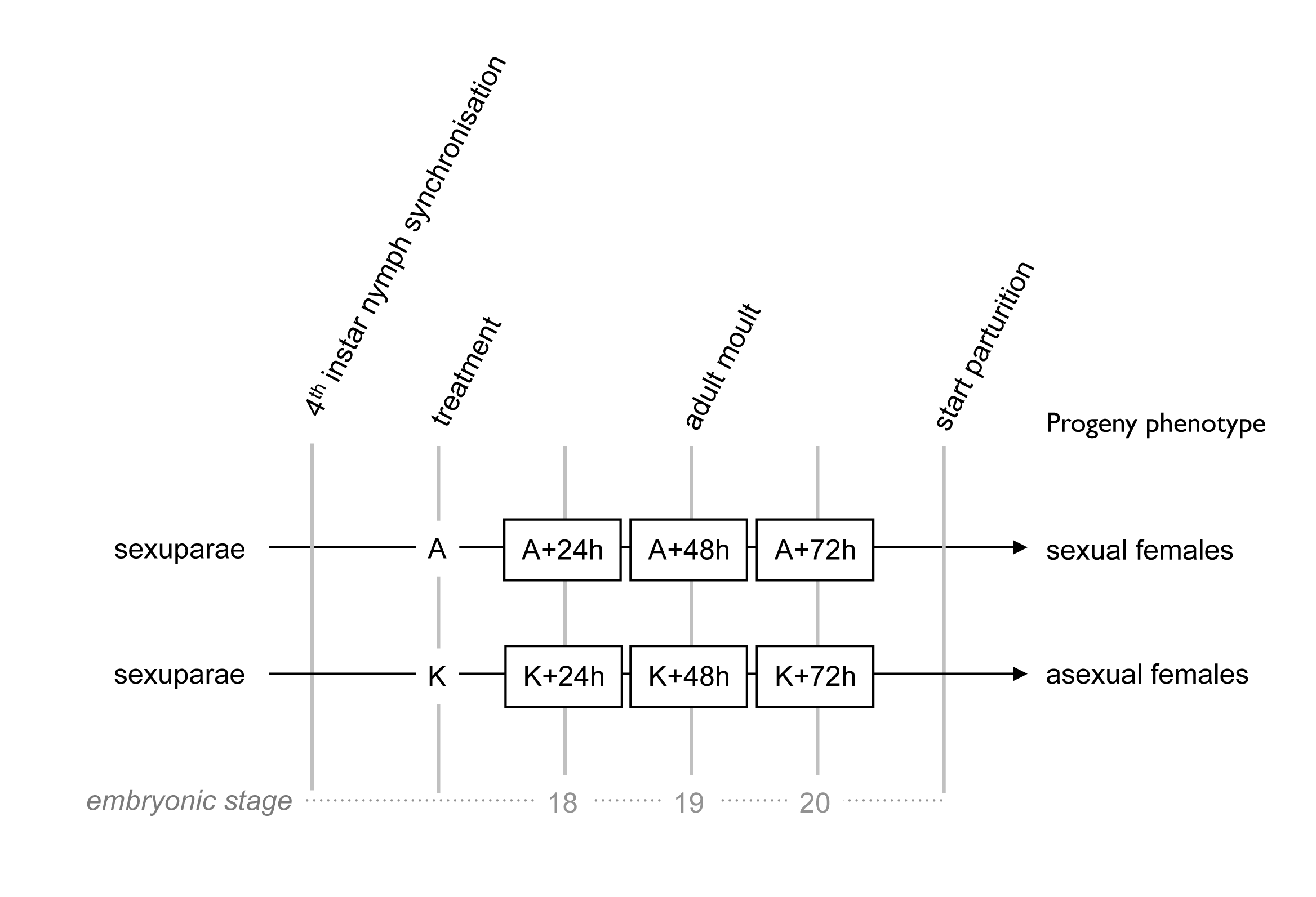

Supplement: Additional file 4 — Experimental design to synchronize the development of pea aphid sexual and asexual embryos. Sexuparae were synchronized at the fourth instar moult in a six hours window. Synchronous sexuparae were randomly separated into two batches; one treated with acetone (A) and one kinoprene (K) 24 h after fourth instar moult. Within each batch, sexuparae were collected 24 h, 48 h and 72 h after treatment, dissected and the 5 most developed embryos were collected for further RNA extraction. [file 1471-2164-13-76-S4.TIFF]
