# Supplementary material for: Sexual and asexual oogenesis require the expression of unique and shared sets of genes in the insect Acyrthosiphon pisum
Source: BMC Genomics. 2012 Feb 15;13:76. doi: 10.1186/1471-2164-13-76 (PMC3313892; doi:10.1186/1471-2164-13-76)
Supplement: Additional file 5 — Primer sequences for cDNA amplification and riboprobe synthesis. Specific PCR primers were designed for each of the regulated transcripts in order to amplify cDNA. [file 1471-2164-13-76-S5.DOCX]

| Transcript | Primer forward | Primer reverse | Tm | Fragment size | Vector |
| --- | --- | --- | --- | --- | --- |
| *ACYPI001842-RA* | AAGTGGCCGATCAGCTAGAA | TTTTCTCAACATAATTATTCTGCACT | 57 | 453 | PCR Cloning Vector pSC-A-amp/kan |
| *ACYPI48336-RA* | GTTAAATTCTCTGCTCTCCGATT | CCACTTTTTCATGTTGAGACATAG | 56 | 866 | PCR Cloning Vector pSC-A-amp/kan |
| *ACYPI000753-RA* | ATCCCGGAAGATGACACAGA | TAATTGGGGGTTCCAATGAA | 58 | 501 | PCR Cloning Vector pSC-A-amp/kan |
| *ACYPI48893-RA* | TCCAGGAAAAGGAGAGGATTT | GCAGACTGCCCTCGATGTAT | 58 | 531 | PCR Cloning Vector pSC-A-amp/kan |
| *ACYPI25088-RA* | TTGGAATGAGCACGACAAAG | TTTTGGCACACAACAAGCTC | 58 | 590 | PCR Cloning Vector pSC-A-amp/kan |
| *ACYPI50630-RA* | CGTTTCATGTATGCGTTTGG | ATGTGCTCAACCGGCTTATT | 58 | 588 | PCR Cloning Vector pSC-A-amp/kan |
| *ACYPI005639-RA* | TCTGAATCTGGAGATGACTCG | GGTGCCTTCTTGGTGACAGA | 58 | 700 | PCR Cloning Vector pSC-A-amp/kan |
| *ACYPI008811-RA* | TTTGTTTCCGGATGCTAAGG | ATTTCCATCGATGCCAGTTC | 58 | 981 | PCR Cloning Vector pSC-A-amp/kan |
| *ACYPI009492-RA* | CCAGAAATGTGTGGCATACG | TGACGTTTGGTCGGAATACA | 58 | 917 | PCR Cloning Vector pSC-A-amp/kan |
| *ACYPI38914-RA* | TGGCCGATAGTCCTCAGTTT | ACCGGATACACATGAATTGC | 57 | 752 | PCR Cloning Vector pSC-A-amp/kan |
| *ACYPI49482-RA* | TGAATCAATACGGTCATGTGC | TTGACGTCCTTCAATGAATCC | 58 | 556 | PCR Cloning Vector pSC-A-amp/kan |
| *ACYPI010082-RA* | GAACAGGTTTCGTGCATGTG | AGTTTGTGGTATGGGCTGGA | 58 | 885 | PCR Cloning Vector pSC-A-amp/kan |
| *ACYPI009671-RA* | GCACCATCCTTGTCCTCTGT | TTCATCAAACGACTGGTTCAA | 58 | 702 | PCR Cloning Vector pSC-A-amp/kan |
| *ACYPI28709-RA* | ATGATGACCAAACGCCAAAT | CGACGGTTGGTCCTGTAGTT | 58 | 876 | PCR Cloning Vector pSC-A-amp/kan |
| *ACYPI31336-RA* | TGCATCCTGCAAATTGTCTT | GCGGTTTCTCATTTCTGGTC | 58 | 427 | PCR Cloning Vector pSC-A-amp/kan |
| *ACYPI39770-RA* | GACAGTGCCACTTCCTTATCAA | CCGAATTCACACAAGCAATG | 58 | 593 | PCR Cloning Vector pSC-A-amp/kan |
| *ACYPI007770-RA* | CTCATCGTCCTGTCTCTTCG | CCTTGCGATTATTCCAGCAT | 58 | 720 | PCR Cloning Vector pSC-A-amp/kan |
| *ACYPI006224-RA* | TGGTATATGGAGCCAGATCG | TGGAGATTGAAAACCTGGAGA | 58 | 709 | PCR Cloning Vector pSC-A-amp/kan |
| *ACYPI007465-RA* | TCTTCCCCGTCTGCTAAAGA | AAACGGCGTTGACATTTCTC | 58 | 894 | PCR Cloning Vector pSC-A-amp/kan |
| *ACYPI54656-RA* | CCTGCGCACTGGAGTAAAAT | AACGTGTCCACCTCTGAAGC | 59 | 591 | PCR Cloning Vector pSC-A-amp/kan |
| *ACYPI005121-RA* | TGGAGTCGAAACAATGAAACC | TTGTCGTTGTCCAAATTTTCC | 58 | 1301 | PCR Cloning Vector pSC-A-amp/kan |
| *ACYPI56634-RA_0* | TATCAAATCCCGCAACAACA | GACCAAAAGCCATGCTCAAT | 58 | 598 | pENTD/D-TOPO |
| *ACYPI007975-RA* | ATGTTTACTAAGCTGGAAAATGAGTA | TGGTCTTATTTCTTTCAATTTAGCTTT | 57 | 491 | pENTD/D-TOPO |
